# Choosing multiple linear regressions for weather-based crop yield prediction with ABSOLUT v1.2 applied to the districts of Germany

**DOI:** 10.1007/s00484-022-02356-5

**Published:** 2022-09-03

**Authors:** Tobias Conradt

**Affiliations:** Potsdam Institute for Climate Impact Research, Telegrafenberg A31, 14473 Potsdam, Germany

**Keywords:** Crop yield modelling, Multiple linear regression, Weather-based yield prediction, Machine learning, Statistical inference

## Abstract

ABSOLUT v1.2 is an adaptive algorithm that uses correlations between time-aggregated weather variables and crop yields for yield prediction. In contrast to conventional regression-based yield prediction methods, a very broad range of possible input features and their combinations are exhaustively tested for maximum explanatory power. Weather variables such as temperature, precipitation, and sunshine duration are aggregated over different seasonal time periods preceding the harvest to 45 potential input features per original variable. In a first step, this large set of features is reduced to those aggregates very probably holding explanatory power for observed yields. The second, computationally demanding step evaluates predictions for all districts with all of their possible combinations. Step three selects those combinations of weather features that showed the highest predictive power across districts. Finally, the district-specific best performing regressions among these are used for actual prediction, and the results are spatially aggregated. To evaluate the new approach, ABSOLUT v1.2 is applied to predict the yields of silage maize, winter wheat, and other major crops in Germany based on two decades of data from about 300 districts. It turned out to be absolutely crucial to not only make out-of-sample predictions (solely based on data excluding the target year to predict) but to also consequently separate training and testing years in the process of feature selection. Otherwise, the prediction accuracy would be over-estimated by far. The question arises whether performances claimed for other statistical modelling examples are often upward-biased through input variable selection disregarding the out-of-sample principle.

**Supplementary Information:**

The online version contains supplementary material available at 10.1007/s00484-022-02356-5.

## Introduction

Weather-based crop yield predictions have a long history; correlations between weather variables and agricultural yields had already been studied in the first quarter of the twentieth century (Meinardus 1901; Hooker 1907; Fisher 1924), and estimating regional yields by multiple linear regressions from time-aggregated weather data has been applied for decades. The full spectrum of potentially yield-relevant meteorological averages in varying seasonal time windows is however rarely scrutinized by the existing models; the same holds for landscape-specific weather response patterns of different crops. The algorithm presented in this article attempts to minimize these gaps by extensive regression testing for automatically selecting the most informative weather aggregate combinations on a per-district basis (however guided by multi-district performance), finally aiming for reliable extrapolations in climate change scenario assessments.

The challenge of weather-based yield regressions is hardly in validating the general approach; it usually works and explains a significant part of the observed yield variations. Numerous studies have just demonstrated that for different crops in different locations around the world (e.g. Ceglar et al. 2016; Nemoto et al. 2016; Schauberger et al. 2017b). It is the details of the implementation which matter and finally decide to what extent the unavoidable prediction errors can be reduced. Even resorting to simple linear regression models leaves the modeller with further decisions galore, frequently too easily taken based on personal beliefs but in fact defining the true challenge of research and development with this class of models.

Optimizing the selection of predictor variables defines the focus of this contribution. The “oldschool approach” still taken by many researchers is largely an expert choice. It might have been guided by selective correlation analyses for preselected candidate variables (González-Fernández et al. 2020; Ji et al. 2019), stepwise regression (Kern et al. 2018; Salehnia et al. 2020), or consideration of crop growth stages for suitable time windows (Butts-Wilmsmeyer et al. 2019; Zhang et al. 2017). In some cases, the selection effort is critically flattened, even if continental climate impact assessments are at stake: (Moore and Lobell 2014, 2015) used temperature and precipitation averages of the growing season as sole meteorological basis for that purpose. The challenge of choosing suitable input variables where optional input data are abundant however paves the way to machine learning. As a non-meteorological example, Gómez et al. (2019) had potato yields automatically fitted to 54 spectral bands and indices of Sentinel 2 satellite images by diverse methods (including generalized linear model, quantile regression, support vector machines, and neural networks). While a massive, automated search for the best predictor features and methodology evades modeller subjectivity, explanations for a certain system behaviour can hardly be given, and an eventual gain in predictive performance remains questionable if there is no strict separation between training and testing data (“out-of-sample”).

By no means, the method presented here can be claimed to be the last word on the subject; however, it systematically optimizes the often disregarded input variable selection process while maintaining the principal approach of multivariate linear regressions. Therefore the code shall be named “Assessing Best-predictive Sets fOr multiple Linear regressions throUgh exhaustive Testing”, in short: ABSOLUT. Version 1.0 lacked the consequent out-of-sample processing in the feature and regression selection parts albeit it was observed for regression testing. This was corrected in v1.1, and the current version 1.2 presented here also excludes overlaps of weather features’ time aggregations in the regression formulas.

The ABSOLUT approach can be counted as a kind of brute force machine learning uncommon to regression-based crop yield prediction: Among the 362 crop forecasting studies published in the years 2004–2019 that were evaluated by Schauberger et al. (2020), there were 258 utilizing regression, and only a few dozen implementing established machine learning approaches most of which were automated neural networks (28 cases) followed by random forests (12). This study should therefore serve as proof of concept for bridging the gap between regression and machine learning approaches which may also be transferable to similar setups, e.g. with panel or nonlinear regression models. The working hypothesis is that regression-based modelling can still compete with machine learning approaches if automated optimizations are applied.

## Materials

### Hard- and software

As ABSOLUT builds on exhaustive searches for optimal feature combinations in linear regressions, parallel execution of the code on a cluster computer using several dozen cores is advisable. A state-of-the-art single PC or notebook would probably need 1–2 weeks for the Germany example given all CPU cores (usually four) are engaged.

The code is written in R (https://www.r-project.org/) and requires version 3.5.1 (used in this study) or newer. It makes use of the extension packages *leaps* (Lumley 2017; v.3.0) and *doMPI* (Weston 2017; v.0.2.2). Suggestions for pre- and postprocessing software can be found in a separate “Directions for use” document placed in the code repository (Conradt, 2021b).

### Input data

Any ABSOLUT application needs a domain divided into spatial subunits, henceforth called districts, for which there are individual crop yield time series and monthly weather data available. A large number of districts (ideally more than 100) and many years with yield data (preferably more than 20) are required for the selection of valid regression feature combinations.

Monthly weather data are needed for each district and should spatially correspond to the agricultural areas within them. The weather data should start at least 1 year before the yield records start and end not earlier than with the growing season of the final year covered by the yield data; otherwise, not all yield information can be considered. In detail, the modeller fixes the last calendar month whose weather data are to be considered for the growing seasons, and the model will evaluate weather–yield correlations in the 12-month periods ending with this month. For example, if there are yield data for the years 1996–2015 and this “cut-off month” is set to May, the weather data must cover the period from June 1995 to May 2015 to utilize the complete yield information.

As it is not necessary to include the very end of the growing season (the actual harvest dates shift between years anyway), a timely cut-off and weather data reaching far enough into the current year can be used for pre-harvest yield forecasts (Schauberger et al. 2017b, regarding the effects of shortened weather input). All data are to be provided in form of ASCII tables; see the example files in the data repository (Conradt, 2021a).

### Specifics of the example application

Germany is a challenging test bed for agricultural modelling due to the heterogeneity of its landscapes and cropping conditions; for a brief geographical description, see section S1 in the Supplement. The spatial basis of the example is Germany’s 401 district-level subdivisions (including the city states of Hamburg and Berlin as single units) as they existed on 1st January 2018. The time frame was principally determined by the district-wise crop data covering the years 1999–2020; yield predictions for 2021 were however possible through more recent weather data. Observed yields for 2021 and further weather data allowing first 2022 predictions became available shortly before publication of this article and were considered in Table 2.

#### Primary data from external sources

The district geometries were taken from the official 1: 1 million digital map of administrative areas, status 1st January 2018, published by the German Federal Agency for Cartography and Geodesy (BKG 2018). Crop yield data are provided by the Statistical Offices of Germany (Statistische 2021b; DESTATIS, 1982ff). National, state, and district level data were obtained for ten crop species in the harvest years 1999–2020. Additionally, district-wise crop growing areas (Statistische 2021a) were obtained for the year 2016 and applied as weighting factors in aggregating the estimated district yields to state and national averages. Monthly weather data were obtained from the Climate Data Centre of the German Weather Service (DWD). Point of departure was their monthly 1-km grids of meteorological variables for the years 1998–2021 (DWD 2021).

The spatial distribution of agricultural areas within the districts has to be taken into account for determining the locally relevant weather conditions, especially for districts including both an agricultural lowland and a mountainous part (Conradt et al. 2016). The 2012 Corine Land Cover data (Copernicus 2020) were used for this purpose. Details of the necessary preprocessing are given in S2.2. Respectively prepared input data as used for the example application have been published (Conradt, 2021a).

## Methods

At its core, the ABSOLUT algorithm applies multiple linear regressions of the form

1 $$y\left(t\right) = \alpha + {\beta }_{0}t +\sum\limits_{i=1}^{d\in \left\{0,\dots ,4\right\}}{\beta }_{i}{w}_{i,t} + \varepsilon$$

for each spatial subunit (“district”) of the target domain. Herein, *y*(*t*) is the yield, i.e. the harvested mass per area, in dt ha^−1^ of a certain crop in the year *t*; *α* is the intercept; and the *β* are the other regression parameters: There is a linear basis trend over time *β*_0 _*t*, and there can be up to four additional terms for aggregated weather variables *w*_*i*,*t*_. Such an aggregated weather variable could, for instance, be the precipitation sum of December, January, and February preceding the harvest in the summer of *t*. These aggregates are henceforth called weather features to avoid confusion with weather variables like temperature or precipitation in general. The closing *ε* is the estimation error to minimize.

The decision to limit the number of weather features *d* to a maximum of four was guided by practical experience with statistical yield model performance. Including more features would not necessarily increase prediction accuracies but come with an even more extensive computational demand for testing myriads of possible feature combinations. Using less features however provides more reliable regression parameter estimations and can be a better choice for certain years and districts. The automatic consideration of different numbers of weather features in Eq. 1 was introduced with v1.2; former versions were hard-wired to *d* = 4.

**Fig. 1:**
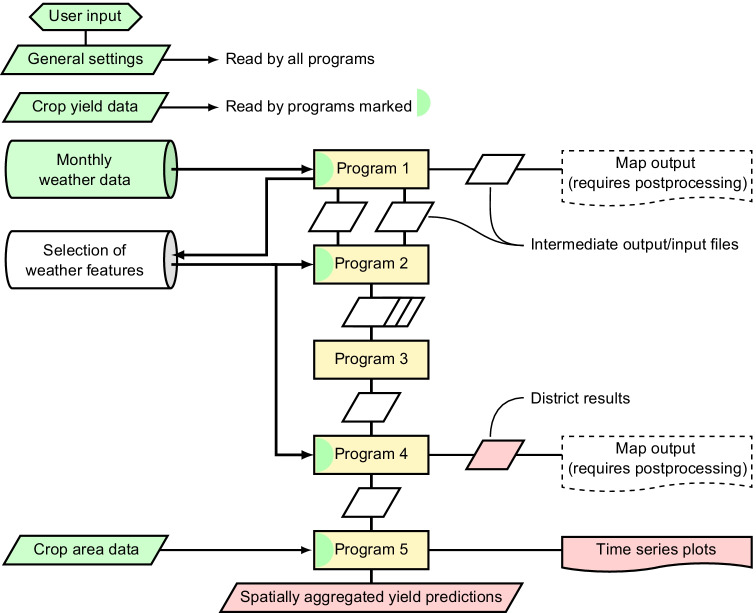
Flowchart of the ABSOLUT programs (yellow boxes) with input, intermediate, and output data. Inputs to be provided by the user are coloured greenish; any other data will be generated in the process. Principal outputs are tinted in red

The R code of ABSOLUT is freely available (Conradt, 2021b) and consists of five programs that have to be run subsequently. The first three of them determine the weather features to be used in the finally selected district models. The sectional workflow, delineated in Fig. 1, is partly owing to different stages of code development and was kept to allow for checks into the intermediate output/input files. The following subsections briefly describe the purposes and main features of the programs; further details are given in S3.

### The five steps of ABSOLUT

#### Program 1: “the prospector”

This initial step is principally exhaustive input feature testing for obtaining best-fitting multiple linear regressions. Although the results cannot be used for predictive purposes directly, it contributes to narrowing the search window for regressions of higher predictive capability. Time aggregates of the chosen weather variables are calculated for periods of 2 to 6 months taken from the 12 months before harvest; these are called weather features. Using all possible start months, this means 11 different 2-month features per variable, ten 3-month features, nine 4-month features and so on, in total 45 features per weather variable, each with one value per year.

The better purpose of program 1 is to subset the pool of possible regression features to those very probably containing predictive power, and this is based on counting the number of occurrences of weather features among the best-fitting regressions. Accordingly, all weather features used in these regressions are sorted along their frequencies of use, and a relevance cutoff is determined based on binomial probabilities—features need to have been selected more often then they would have occurred by pure chance with 99.9% probability; the number of features above this threshold is henceforth denoted *q*. Details of the calculation are explained with the case study example below.

Note that all calculations made in this and the following programs are done separately for each target year, so that any feature selection and prediction is made solely based on information from other years plus the meteorological data of the target year. Only for current and future year projections (scenarios), all available data from the past are utilized.

#### Program 2: “the workhorse”

Program 2 effects the highest computational burden, using parallelisation is strongly recommended. Again, possible input feature combinations are investigated for their predictive power in multiple linear regressions according to Eq. 1, but the weather features are now chosen from the target year-specific preselections of significant features provided by program 1. Consequently, only predictions for these years whose yield data are censored from the regression equations (leave-one-out) are calculated. What is demanded here is an evaluation of the predictive skill of the input feature combinations, not just single features. This is done by Pearson correlations (*r *values) between reported yields and the out-of-sample yield predictions from the regressions.

#### Program 3: “the gold pan”

The logically following task addressed by this program is determining the optimal regression model for each spatial subunit. Simply choosing the feature combinations separately for each district from top of their local *r *ranking (“local heroes”) could however be misleading because a single yield time series estimation provides rather insufficient validation; any chosen combination should be cross-validated by above-average performances in many districts.

The solution currently implemented (“global and local heroes”) merges globally best performing combinations (highest average *r *values) with those working exceptionally well in smaller subsets, down to 10% of the districts. The idea behind is to account for special conditions in certain landscapes. The locally best-performing regression out of this third selection is finally implemented for each district.

#### Programs 4 and 5: “crucible and mould”

What remains is using the selected regression equations for yield prediction in the respective target years; this is done by program 4. Program 5 aggregates the district yield predictions by weighted averaging to predictions for the full modelling domain. Spatial aggregation provides higher prediction accuracy due to mutual error compensation among noisy district results.

### Setup of the Germany application

Three principal weather variables were selected: average temperature, precipitation, and sunshine duration. Temperature governs the physiological processes in plant growth and fertility. Sunshine is used as proxy for radiation, the energy source for photosynthesis. Precipitation and radiation (the main driver for evapotranspiration) are finally decisive for water stress. There are actual radiation data available, but sunshine duration is measured at more locations and regularly delivered better results. Applying ABSOLUT to other world regions and crop species may require other meteorological variables for optimal results, and data availability has always to be considered. Even non-meteorological variables would be acceptable, but here, we focus on weather effects. The minimum of yield data per district had been set to 17 because the example dataset was limited to 22 years (1999–2020), and a higher requirement would have excluded many districts with incomplete observations.

The primary test crop was winter wheat, and the last month for weather input before each year’s harvest (typically in July or August) was set to June. For the secondary test crop silage maize, the weather input season was set to end by August; maize harvest may occur late in the year but growth stagnates in autumn.

## Results

### Observations along the workflow for winter wheat

#### Running program 1

With the three weather variables 3 · 45 = 135 weather features were generated. This meant $$\left(\begin{array}{c}135\\ 4\end{array}\right) = 13\;232\;835$$ different regressions per district and target year to be tested which required a couple minutes using 24 CPUs in parallel.

There is a stark difference between the goodness-of-fit of the top-ranked regressions applied to the same data that was used for selecting them and their performance in validation mode, i.e. with the observed yield value of any single year to predict censored from the input (out-of-sample validation across all target years). The respective *r*^2^ values for winter wheat averaged over all district models are 0.878 and 0.115, Fig. S2 shows maps of the the spatial distributions. It is clear that the regressions selected in this step cannot be immediately used for predictions.

**Fig. 2:**
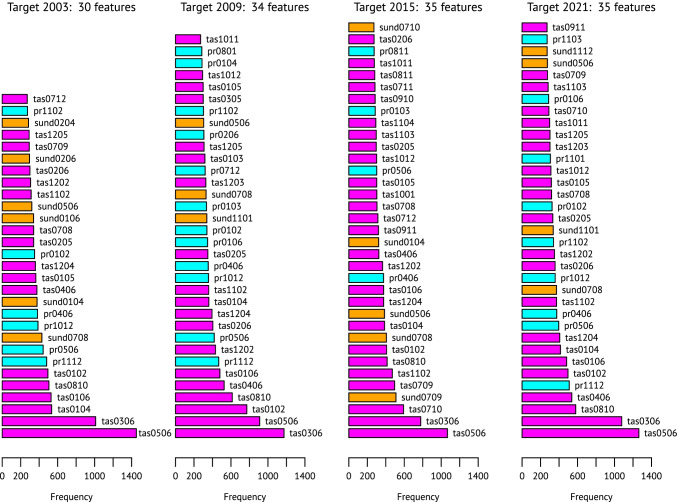
Frequencies of weather features found significantly often in the regression equations optimally describing observed winter wheat yields in the district-level administrative units of Germany. Results for four selected target years whose yield data have been omitted from the input data available for 1999–2020. The feature names consist of variable acronyms (tas = temperature, pr = precipitation, sund = sunshine duration; also signalled by the colours) and two-digit numbers of the start and end months of their time aggregation

Which weather features do however appear significantly often in these regressions, each target-year collection including the top 23 per district? The algorithm requires that the number of occurrences exceed a frequency expected by pure chance with 99.9% confidence. If there were pure noise in the data, each of the 135 features originally provided would turn up with a constant probability of $p = \frac{4}{135}$ per regression sample. With a finite number of samples their frequencies follow a binomial distribution. In the winter wheat case, there were *n* = 7498 samples (326 districts times 23) per target year; thus, the expectation value for any weather feature in the noninformative case would be *E*(*x*) = *np* = 222.163 occurrences with an expected standard deviation of $E\left(\sigma \right) = \sqrt{np\left(1-p\right)} = 14.683$. The number of occurrences not to be exceeded in 99.9% of the cases would be *P*_999_(*n*,*p*) = 269. Depending on the actual frequencies in the separate target-year outputs, between 29 and 36 weather features were selected in the winter wheat case; Fig. 2 shows the selections and frequencies for four target years.

The average temperature towards the end of the growing season (temperature aggregates for May and June and March to June) stick out clearly for each target year; this is in full agreement to the often described temperature sensitivity of wheat during and after anthesis (Akter and Islam 2017; Farooq et al. 2011; Schauberger et al. 2017a). Consequently, the majority of the aggregates shown in Fig. 2 are temperature averages (magenta), while less than a quarter are precipitation depths (cyan) and only 18 out of 134 are sunshine durations (orange). The frequency of selections depends strongly on which year was omitted from the input data, but there are also quite stable and interesting patterns: For example, sunshine duration in July and August (in the pre-harvest years, before sowing!) is selected for every year shown in Fig. 2 and in 21 of the 23 target years considered in total.

#### Running programs 2 and 3

The 23 target-year specific output tables of program 2 have between *A*_2004_ = 4050 and *A*_2000_ = 16 216 lines (below the header) for all possible input variable combinations and 326 columns representing the districts for which enough yield data were available. Negative correlation coefficients could be found in 28.3% of the table cells. The negative extremes are near-perfect anticorrelations; eight target years had *r*_min_ <  − 0.97.

Given the number of 22 out-of-sample regression estimates behind every correlation coefficient, the computational demand is significant. Running program 2 on 112 CPU cores took about 23 h for winter wheat. Calculations for other crops led to fewer pre-selected input weather features and could be done in a few hours, though. It should also be noted that the major numerical effort is done with program 2, the remaining code parts only take a couple minutes to complete on a single CPU.

Even for the generally best performing feature combinations, the individual per-district correlations between out-of-sample predictions and observed yields are rather noisy. Consequently, 258–290 different combinations make up the individually best-performing regressions for the 326 districts considered in the winter wheat case, and their average correlations of 0.717–0.766 still deteriorate when used for predictions based on new data.

The input feature combinations leading to the highest out-of-sample average correlations (averaged over all spatial subunits) are determined by program 3. The top-ranked combination for each target year is listed in Table 1: Note that tas0506 is frequently included, while tas0306 does not occur at all here despite the fundamental positioning of both features in Fig. 2.

**Table 1:** Target year-specific combinations of input weather aggregates performing best across all districts and the average Pearson correlation of their out-of-sample predictions in the winter wheat example for Germany; output of program 3. The input feature tas0506 is bolded to highlight its many occurrences

Target	Input weather aggregtes				*r̄*
1999	**tas0506**	sund0506	tas1202	tas0811	0.465
2000	tas0102	**tas0506**	tas0710	pr1102	0.475
2001	**tas0506**	sund0506	tas1202	tas0811	0.466
2002	tas0102	tas0810	tas0406	pr1102	0.434
2003	**tas0506**	sund0506	tas0104	sund0104	0.441
2004	pr0102	**tas0506**	sund0506	tas1202	0.455
2005	**tas0506**	sund0506	tas1202	tas0710	0.467
2006	**tas0506**	sund0506	tas1202	pr1103	0.460
2007	tas0102	**tas0506**	pr0506	pr1102	0.453
2008	tas1011	**tas0506**	tas1202	pr1102	0.484
2009	tas1011	pr0102	**tas0506**	tas1202	0.463
2010	**tas0506**	sund0506	tas1203	sund0104	0.504
2011	tas0102	**tas0506**	tas0710	pr0902	0.488
2012	**tas0506**	tas0710	pr0811	tas1203	0.495
2013	**tas0506**	pr0506	tas1202	pr1202	0.444
2014	tas0102	**tas0506**	tas0710	pr0812	0.446
2015	**tas0506**	sund0506	tas1202	tas0711	0.474
2016	tas1011	pr0102	**tas0506**	tas1202	0.524
2017	tas0102	**tas0506**	tas0710	pr0801	0.482
2018	tas0102	**tas0506**	tas0710	pr0812	0.464
2019	pr0102	**tas0506**	sund0506	tas1202	0.474
2020	**tas0506**	sund0506	tas1202	tas0710	0.478
2021	**tas0506**	sund0506	tas1202	tas0710	0.473

The “global and local heroes” selection was finally applied to determine the target-year specific sets of predictors; see S4 for the analysis of the alternative selection methods. For most years, 12–16 combinations were actually applied, the maximum was *c*_2004,2005_ = 19, and the minimum *c*_1999_ = 9. These combinations contained 12–17 different weather features, and the correlation averages of the so determined district models were in the range of 0.572–0.650; these numbers are a more realistic indication of the expectable prediction performance.

#### Running programs 4 and 5

**Fig. 3:**
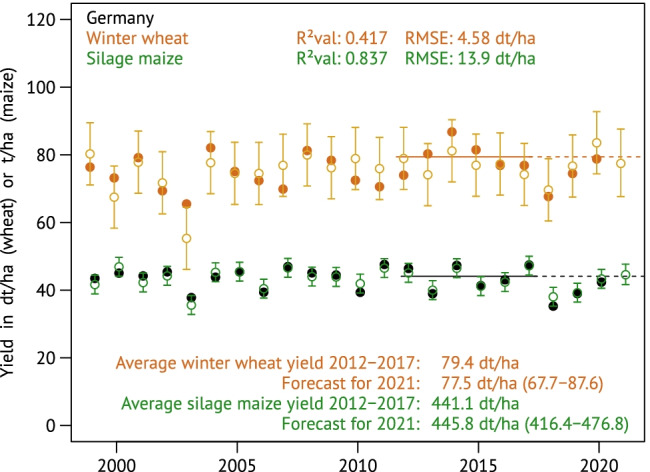
Winter wheat and silage maize yields in Germany according to the official statistics (DESTATIS, 1982ff, solid dots) and their out-of-sample predictions with uncertainty intervals. The uncertainty bars on the hindcasts extend to ± 1.96 times the sample standard deviation of the prediction errors and should therefore cover 95% of the observed yields. For 2021, the actual prediction interval is shown; this is slightly wider due to the uncertainty of the estimation and centred on the sample mean of prediction errors which may slightly deviate from zero

Program 4—utilizing the out-of-sample-determined district-specific regressions for the out-of-sample yield predictions—loops through all districts within a minute. Their spatial time-series aggregation towards Germany’s national winter wheat yields, computed by program 5, is shown in Fig. 3 together with the results for silage maize. In contrast to the intermediate aggregation for the federal state of Saxony (Fig. S3), the national estimates for winter wheat yields expose higher noise but reduced errors (*R*^2^_val_ = 0.417, RMSE = 4.58 dt ha^−1^), while the spatially aggregated silage maize modelling shows remarkable accuracy (*R*^2^_val_ = 0.837, RMSE = 13.9 dt ha^−1^).

### Prediction performance

#### Regional performance for 2018 silage maize yields

**Fig. 4:**
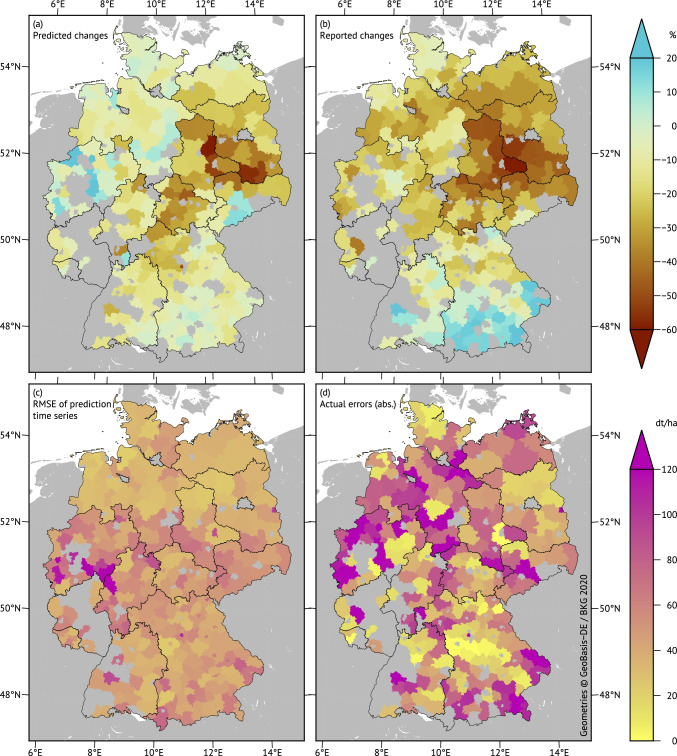
Spatial characteristics of the silage maize yield prediction for the drought year 2018. **a** Predicted yield changes compared to the average district yields of the years 2012–2017. **b** Observed changes according to the official statistics. **c** Root-mean-square errors (RMSE) of all out-of-sample district yield predictions for the years 1999–2020. **d** Absolute values of prediction errors for 2018. **a** and **b** show relative deviations in percent (upper scale), **c** and **d** refer to absolute deviations in dt ha^−1^ (lower scale)

Using district regressions provides not only a basis for aggregate results but can also help identify spatial patterns despite the higher noise in single district results. In contrast to major agricultural zones of the world like the North China Plain or the US corn belt, Germany is characterized by a high diversity of soil landscapes forming a distinct pattern of high and low yield regions (Hennings 2013; Kruse 2016); hence, the ability to predict spatial yield patterns should be determined from relative changes as shown in the upper map panels of Fig. 4.

The silage maize harvest forecast for the drought year 2018 was chosen as an example due to extreme yield losses concentrating in Eastern Germany. The S5 section in the Supplement presents respective maps for the 2019 yield changes of winter wheat, sample statistics of both cases, and observations from the error compensation for spatial aggregates.

Despite the noise, the maps of the predicted and observed relative yield changes in districts shown in Fig. 4 (a and b) show a general similarity of spatial patterns. There are regional misses especially in north-western parts of the country and along the southern border, but the centre of gravity of the strongest yield losses could correctly be located. Panel (c) gives an impression of the regional distribution of prediction power through absolute RMSE values calculated from the complete record of 1999–2020 out-of-sample prediction errors. In general, the method works fine in Northern Germany and some parts of the south, but has some issues in western to central areas. The actual 2018 forecast errors (d) were much larger in many districts including those with comparably small RMSEs. It may be assumed that the training period (1999–2017) did not contain enough reference drought years; in fact, only 2003 might have been comparable to some extent.

#### Yield predictions for Germany

There are at least two institutions regularly publishing crop yield forecasts or estimates: the German Federal Statistical Office (Statistisches Bundesamt, DESTATIS) and the Joint Research Centre (JRC) of the European Commission with their MARS (Monitoring Agricultural ResourceS) activity. The DESTATIS reports (DESTATIS, 1982ff) with national and federal states’ estimates are based on extensive field monitoring, on-site observations during growth and harvest by farmers and travelling experts. The MARS forecasts utilize a number of sources and predictors but seem to largely rely on remote sensing. National aggregate predictions are released via monthly bulletins (MARS 1993ff).

Yield predictions for crops harvested in June or July (cereals or rape) can usually be computed in the beginning of July as soon as the monthly weather data for June are available. For silage maize or sugar beets, the August weather data should be completed. Both conditions were constantly observed for the examples presented. Hence, the preliminary national crop yield estimations of DESTATIS, regularly published in the beginning of August and at the end of September, are used for comparison. Only the winter barley estimates are released in between (which explains their small deviations from the final figures), and DESTATIS does not provide early indications at all for sugar beet yields. For MARS, the annual issues 7 (typically released at the end of July) and 9 (typically released in mid-September) are the seasonal counterparts to compare with. Table 2 compares the official national yields with the different predictions for five crops in the years 2018–2021 and also shows some forecasts for 2022.

**Table 2:** National average yields for various crops: comparison of official statistics (yield) to near-harvest predictions of ABSOLUT, the German Federal Statistical Office (DESTATIS, 1982ff), and the European Commission’s Joint Research Centre (MARS, 1993ff)

	Yield	ABSOLUT		DESTATIS		MARS	
		Prediction	Error	Prediction	Error	Prediction	Error
Year	dt ha^−1^	dt ha^−1^	dt ha^−1^	dt ha^−1^	dt ha^−1^	dt ha^−1^	dt ha^−1^
Winter wheat		*07–09 July*^*a*^		*02–03 August*		*22–27 July*	
2022‍		76.3		71.0		74.5^*b*^	
2021	73.5	77.5	+ 4.0	77.5	+ 4.0	79.3^*b*^	+ 5.8
2020	78.8	83.3	+ 4.5	71.9	− 6.9	76.0^*b*^	− 2.8
2019	74.5	79.6	+ 5.1	73.0	− 1.5	77.1^*b*^	+ 2.6
2018	67.7	71.2	+ 3.5	66.4	− 1.3	71.6^*b*^	+ 3.9
Winter barley		*07–09 July*^*a*^		*24–29 August*		*22–27 July*	
2022‍		67.1				71.9	
2021	71.6	72.3	+ 0.7	71.9	+ 0.3	70.5	− 1.1
2020	67.3	75.3	+ 8.0	67.5	+ 0.2	68.8	+ 1.5
2019	72.2	73.4	+ 1.2	72.1	− 0.1	71.3	− 0.9
2018	60.6	58.8	− 1.8	60.8	+ 0.2	63.5	+ 2.9
Rye		*07–09 July*^*a*^		*02–03 August*		*22–27 July*	
2022‍		49.7		52.2		52.0	
2021	52.7	54.8	+ 2.1	58.4	+ 5.7	57.1	+ 4.4
2020	55.2	54.8	− 0.4	51.7	− 3.5	52.5	− 2.7
2019	50.9	47.5	− 3.4	51.7	+ 0.8	52.8	+ 1.9
2018	42.1	46.0	+ 3.9	42.7	+ 0.6	44.6	+ 2.5
Rape		*07–09 July*^*a*^		*02–03 August*		*22–27 July*	
2022‍		33.5		33.2		35.1	
2021	35.1	36.1	+ 1.0	36.7	+ 1.6	37.6	+ 2.5
2020	36.9	37.7	+ 0.8	32.8	− 4.1	32.7	− 4.2
2019	33.1	34.9	+ 1.8	33.8	+ 0.7	34.7	+ 1.6
2018	30.0	29.9	− 0.1	28.8	− 1.2	30.0	± 0.0
Silage maize		*07–09 September*^*a*^		*22–26 September*		*14–20 September*	
2022‍							
2021	472.3	445.8	− 26.5	451.0	− 21.3	457.0	− 15.3
2020	423.9	435.3	+ 11.4	410.3	− 13.2	400.0	− 23.9
2019	390.0	383.1	− 6.9	383.7	− 6.3	394.0	+ 4.0
2018	352.9	387.9	+ 35.0	342.7	− 10.2	361.0	+ 8.1

^*a*^Hypothetical release dates for ABSOLUT predictions assuming 3 work days for calculations after publication of the meteorological grids

^*b*^The winter wheat figures given for MARS have been obtained by adding 0.6 dt ha^−1^ to their original soft wheat prediction

### Weather input of Gornott and Wechsung

Experiments with district-based crop yield prediction in Germany through multiple regression using weather aggregates had already been presented by Gornott and Wechsung (2016). In contrast to the algorithm presented here, only year-on-year (YoY) changes were considered saving the explicit estimation of an underlying linear trend. The study investigated different options to couple the coefficients of the district models (panelling), an approach further pursued with cluster analysis (Conradt et al. 2016). The nearest equivalent to ABSOLUT are therefore the independent district regressions of Gornott and Wechsung (2016), called there “separate time series models” (STSMs), and the most fundamental difference is that all STSMs used the same set of input variables—predefined per crop—while ABSOLUT searches for some optimal combinations.

**Fig. 5:**
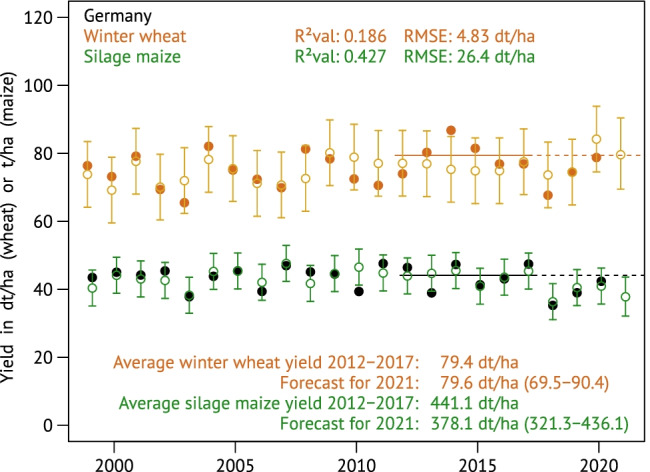
Winter wheat and silage maize yields in Germany according to the official statistics (DESTATIS, 1982ff, solid dots) and their out-of-sample predictions calculated from the five weather aggregates used by Gornott and Wechsung (2016). Uncertainty and prediction intervals calculated as in Fig. 3

How powerful are the predefined input variables in terms of prediction accuracy compared to the combinations drawn by ABSOLUT? To answer this question, the district regressions were charged with the weather variables originally used by Gornott and Wechsung (2016); implementation details are given in S7. The time series for national yield predictions calculated from the Gornott and Wechsung weather aggregates are shown in Fig. 5. Compared to the result of the ABSOLUT algorithm in Fig. 3, the lower accuracy is evident; the shares of explained interannual winter wheat yield variability dropped from 41.7% to mere 18.6%. Note that the wheat predictions resemble the observed ups and downs predominantly in the first half of the time; from about 2010 onwards, there is hardly any correlation any more. The coefficient of determination for the national silage maize yield predictions reaches at least 42.7%, while Gornott and Wechsung (2016) reported 50% for their prediction of interannual changes. This is however clearly below the 83.7% obtained with ABSOLUT. Maps showing the spatial goodness-of-fit distributions can be found in the Supplement (Figs S5 and S6).

## Discussion

### Performance in comparison to previous studies and official yield predictions

The first lesson learned was that the excessive testing and optimization of regressor combinations consumes degrees of freedom, thus predictive power, just like the estimation of many coefficients within the multiple regressions. The solution was to require significant above-average performances in many districts for qualifying combinations of weather aggregates (input features) as predictors: Their performance exceeded the results obtained with pre-defined weather features used in precursor studies (Gornott and Wechsung 2016; Conradt et al. 2016).

Considering the fact that the ABSOLUT results can be obtained 2–4 weeks in advance to those of the other sources, the quality of its predictions of national yield averages is in the same league with DESTATIS and MARS. The ABSOLUT regressions produced tendentially more overpredictions, probably due to uncaptured drought effects (especially soil drought). The explained shares of yield variations are in accordance with the literature: Global studies assessing the relative impact of weather factors on crop yield variations (Frieler et al. 2017; Schauberger et al. 2017b) give typical ranges of 50–60% for wheat and maize yields of main producer countries; for wheat in the USA, only 30–40% were reported as well. A careful assessment separating the impacts of farm management and weather effects on wheat yield variations across Germany (Albers et al. 2017) found average shares of 43% of the variations caused by the weather and 49% owing to management. While non-meteorological factors like irrigation status, fertilizer price, and general farming conditions are much more decisive in developing countries (Assefa et al. 2020), national aggregate yields of staple crops in Europe may depend even more on weather than previously assumed (Agnolucci and De Lipsis 2020). To tap the full potential of weather-based yield modelling, meteorological extremes (heat waves, storm precipitation) need however also to be considered; by using only time aggregations over several months, this is not possible.

Regarding possible COVID-19 effects in the official 2020 and 2021 yield data, farmers’ cropping operations had been done as usual in Europe (no COVID restrictions for single-driver machines). Only pandemic-induced micrometeorological effects not reflected in the weather data like sunshine intensity and alterations in air chemistry (less NO_2_, more O_3_) caused by reduced air pollution (Skirienė and StasiškienėŽ 2021, Torkmahalleh et al. 2021; Silva et al. 2022) may have affected the observed yields. However, no literature specifically devoted to lockdown effects on plant growth could be found, so these effects are probably hardly traceable.

### An over-confidence trap in statistical modelling

The most important improvement of ABSOLUT v1.2 over its predecessor v1.0 is that not only the parameter estimations used for prediction are solely based on weather–yield relations in other years than the the target year (out-of-sample) but also the search for the weather features to be applied. Originally, the feature combinations for the district regressions were fixed once and for all based on the full dataset. The biased *R*^2^_val_ indications of v1.0 reached more than 0.8 for the national winter wheat yield time series, and silage maize results were even breaking 0.9.

The performance indications have now been corrected by the consequent separation of training and testing data for all aspects of feature selection and parameter estimation. Forecast and scenario outputs were hardly affected by the correction—all program versions use all available data from the past for predictions beyond the coverage of the observed yield time series. How much the performance measures had been upward biased revealed the enormous information content hidden in the selection of input feature combinations.

This is important for any related kind of statistical modelling: Especially if the selection of input variables is less freely adaptive but guided by expert knowledge, it may happen quite often that the resulting model performs seemingly well, but only in the environment in which it was developed. With the recent input data, the historical prediction performances of Gornott and Wechsung (2016) (cf. also Conradt et al. 2016) could be reconstructed to some extent for silage maize but hardly at all for winter wheat. An interpretation is that the formerly observed correlations between uniformly defined weather variables and wheat yield deteriorate under climate change and influential weather variables become gradually replaced by others (to which the ABSOLUT algorithm will automatically adopt). Given the rather abrupt loss of correlation in Fig. 5 after 2010, the final year considered by Gornott and Wechsung (2016), it can as well be assumed that their input variable selection was (unconsciously?) guided by its performance in the historic environment, and any data from outside the original time window would spoil the original correlation even without climate change.

The question remains, to what extent information absorbed in the model setup process and henceforth contained in model structures makes overconfidence in predictions from new input data a common issue, not only in weather-based crop yield modelling. In recent years, machine learning algorithms gained popularity in crop modelling with seemingly better results than multiple regression modelling (Cai et al. 2019; Cao et al. 2021; Leng and Hall 2020; Zhang et al. 2020; Bouras et al. 2021). However, practically all of these studies have in common that an initial selection of predictor variables was made using all available data (and often simple tools like Pearson correlations) before the advanced methods were applied.

### Improvement potentials and development opportunities

There are two critical spots of the present version of the algorithm which are at the same time opportunities for improvements with future revisions: The first one is the assumption of a linear base trend of yields independently estimated for each spatial subunit. This allows straightforward prediction of absolute yields instead of relative changes (a major difference to Gornott and Wechsung 2016, and Conradt et al. 2016), but might be oversimplistic: After several decades of technological progress with ever increasing yields, there are stagnations reported for different crops and world regions (Chen 2018; Schauberger et al. 2018; Mehrabi and Ramankutty 2019). Already existing methods like the stochastic trend separation by Agnolucci and De Lipsis 2020) highlight the potential for improvement.

The second area of concern is the final selection of independent regressor variables, i.e. weather aggregates, for each spatial subunit. The general challenge is about finding the optimum balance between a high number of multi-site confirmations of predictive power and the flexibility needed to adopt to smaller regions demanding alternative combinations for more exact predictions. Perhaps spatial clusters of predictors should be explicitly considered similarly to what has been done for parameter values (Cai et al. 2014; Conradt et al. 2016). Finally, there is also no stability of the weather–yield relations over time, probably caused by nonstationarity of meteorological variables in the context of climate change: Correlation shifts have been observed by Trnka et al. (2016) or Ceglar et al. (2020) which calls for additional flexibility.

This flexibility could also be connected to shifts in the phenological calendar, a well-known effect of climate change (Racca et al. 2015; Zhang et al. 2022). Shifting growth stages could probably be considered by shifting time windows for weather feature aggregation which in turn would require a finer time resolution of the weather input data, e.g. decadal instead of monthly data.

## Conclusions

It could be demonstrated that the ABSOLUT algorithm, already in its present stage of development, is capable of explaining significant shares of the national yield variations of major crops in Germany solely based on weather variables. Given the near real-time availability of German weather data, early in-season yield predictions are possible with accuracies comparable to official national and EU forecasts.

Probably the most important finding was the “overconfidence trap” for any kind of regression modelling with expert-guided regressor selection: As the choice of regressors contains a similar amount of information as the parameter values do, it is very easy to unvoluntarily violate the principle of independent model training and testing. Many performance figures given in the literature for statistical yield models may be positively biased for that reason. The algorithm presented here tries to escape this trap by objectivizing the regressor selection; however, some basic choices for relevant meteorological variables still remain with the modeller.

Primarily developed for demonstrating the feasibility and principal advantage of semi-automatic regression feature selection, ABSOLUT offers many potentials for improvements. Among these is the capability to capture nonlinear long-term yield trends or a better way to balance temporal and spatial correlations in the input data. A weak spot shared with other regression models is the time aggregations blinding the model for exceptionality and effects of (short-time) weather extremes which become more frequent under climate change. Consequently, related questions about the impact of climate change on food security underline the need for further research into this field.

## Supplementary Information

Below is the link to the electronic supplementary material.

Supplementary file1 (PDF 5028 KB)

## Data Availability

The input data needed for the example application to Germany consist of crop yields and cropping areas in administrative regions, district-level monthly weather data, and a control file. They are publicly available at 10.5281/zenodo.5625774 (Conradt, 2021a).

## Code availability

The model code, consisting of five R programs, is publicly available at 10.5281/zenodo.5789350 (Conradt, 2021b).
