# Supplementary material for: Choosing multiple linear regressions for weather-based crop yield prediction with ABSOLUT v1.2 applied to the districts of Germany
Source: Int J Biometeorol. 2022 Sep 3;66(11):2287–300. doi: 10.1007/s00484-022-02356-5 (PMC9440329; doi:10.1007/s00484-022-02356-5)
Supplement: Supplementary file 1 — Supplementary file1 (PDF 5028 KB) [file 484_2022_2356_MOESM1_ESM.pdf]

Supplement

to

Choosing multiple linear regressions for weather-based crop yield prediction with ABSOLUT v1.2 applied to the districts of Germany

Tobias Conradt, conradt@pik-potsdam.de

Potsdam Institute for Climate Impact Research, Potsdam, Germany

International Journal of Biometeorology

Contents

S0 What is ABSOLUT v1.2 ? 2

S0.1 The name of the game 2

S0.2 Version history 2

S1 Germany as test bed for agricultural modelling 2

S2 Requirements for running ABSOLUT 3

S2.1 Hard- and software 3

S2.2 Preprocessing and actual input data 3

S3 Workflow along the five programs of ABSOLUT 4

S3.1 Program 1 – “the prospector” 6

S3.2 Program 2 – “the workhorse” 7

S3.3 Program 3 – “the gold pan” 8

S3.4 Programs 4 and 5 – “crucible and mould” 9

S4 Sensitivity to the selection alternatives in program 3 10

S5 Regional performance: Distibutions of district results 12

S5.1 Silage maize 2018 12

S5.2 Winter wheat 2019 12

S6 Error compensation in spatial aggregates 14

S7 Details of the Gornott and Wechsung implementation 15

References 17

S0 What is ABSOLUT v1.2 ?

The five R programs which make up ABSOLUT contain a staged modelling algorithm for crop yield prediction. It is exclusively applicable for agricultural landscapes with distributed weather and yield observations, e.g. the administrative subdivisions of a country for which respective statistical and meteorological data are available.

At the core of ABSOLUT are multiple linear regressions, one per district, estimating the annual yields of a given crop from a linear time trend and up to four multi-month aggregates of meteorological variables from before the individual harvests. In contrast to other regression approaches, the combinations of weather aggregates are not pre-defined but exhaustively searched and selected by their out-of-sample prediciton performance.

S0.1 The name of the game

The acronym ABSOLUT stands for "Assessing Best-predictive Sets fOr multiple Linear regressions throUgh exhaustive Testing" – the idea behind has been described above. It also hints to the *absolute* value yield predictions, in contrast to setups using relative yield differences the author had worked with before (Conradt et al. 2016).

S0.2 Version history

This document is about version 1.2, released in December 2021. Improvements over the preceding v1.1 are the exclusion of overlapping weather features of the same meteorological variable and the inclusion of regressions with less than four weather features, down to the pure time trend.

Version 1.1, released in October 2021, removed a major inconsistency regarding the out-of-sample approach:

Version 1.0 was available since January 2021. It had been described in a preprint (Conradt 2021c), but the impressive hindcast performances presented there dramatically overestimate the expectable prediction accuracy due to partially violated out-of-sample conditions. While regression testing was correctly made with separate training and testing data (the latter being single years, "leave-one-out"), the input feature combinations of the district regressions were selected based on the entire data set. (Actual yield predictions in operational applications or scenario modelling results are hardly affected by this error, though.)

S1 Germany as test bed for agricultural modelling

An area of 117 630 km^2, a third of Germany's land surface, was used as cropland in 2016 (DESTATIS 2017). With annual production rates of approximately 20–25 million tons of winter wheat and 80–100 million tons of silage maize Germany ranks among the top staple crop producers of the European Union. The croplands are dispersed across all parts of the country only seriously intermitted by mountainous areas and urban agglomerations.

The moderate climate allows autumn sowing for many cultures; nowadays average January temperatures range above 0°C in most parts of the country, and snow accumulation is usually limited to the higher elevated regions (> 500 m amsl). Typical summer temperature averages are just below 20°C, but more than 30°C are regularly observed on the hottest days, and early heat periods can be harmful for cereals. A gradient of continentality is associated with more pronounced temperature extremes in the eastern and southern parts of the country, but elevation is more important for the local climate, especially for precipitation: The national long-term average of 789 mm year^{-1} (DWD 2020) is geographically differentiated between the lower parts of Eastern Germany receiving less than 600 mm year^{-1} and coastal and higher elevated regions in the west where 1000 mm year^{-1} and more are not an exception. Extreme rainfall events and hailstorms which can completely destroy plant cultivations locally have been observed more frequently in the south.

The most important factor for a variety of different cropping conditions is however the highly differentiated soil landscape: region-specific orogenetic and erosive processes including a number of glacial formations in Northern Germany and near the Alps provided the full spectrum of substrates from clays to sands and peat soils (cf. European Soil Bureau Network 2005, Richter et al. 2007, Hennings 2013). This landscape challenge and a decent availability of weather and yield observations qualify Germany as test environment for any kind of weather-related crop yield modelling.

S2 Requirements for running ABSOLUT

S2.1 Hard- and software

The R software (https://www.r-project.org/) is required in version 3.5.1 or newer, and the following packages must be installed to run the code as provided (version numbers indicate the versions used by the author):

leaps Exhaustive search for regression subset selection (3.0) and

either *doMPI* Parallel computing via MPI (0.2.2) which also loads *foreach* (1.4.4)

or *foreach* on its own to run the `doParallel` command in case the hardware does not support MPI.

A potentially useful package for preprocessing weather data is *ncdf4* (1.16.1) which can handle NetCDF grids. It however requires a NetCDF system library.

Further program recommendations for preprocessing and output visualization are the open source programs GDAL (3.0.4), GRASS (7.8.3), and GMT (6.0.0).

S2.2 Preprocessing and actual input data

Preprocessing was required due to a history of district reorganisations (major ones took place in Eastern Germany in 2007, 2008, and 2011). For some federal states the original yield table was still based on historical district geometries, these were resampled to the 2018 administrative geometries. Special care was taken where old districts merged into new ones differed in their agricultural area shares or the delineation of new districts was only partly based on former boundaries.

Spatial resampling of weather data to the agricultural areas within districts is usually required for any application of ABSOLUT. One possibility would have been interpolating weather station data to area centroids (cf. Conradt et al. 2016), but here weighted averages of the DWD grid cells overlapping the croplands were used. First, 401 binary raster maps (one per district) were made with cell values of one indicating cropland (CLC code 211 = non-irrigated arable land) located within the district and zero elsewhere. These maps were then resampled by averaging the cell values to the coarser 1-km grid of the DWD weather data in another map projection. The output grids were interpreted as weight matrices for resampling the gridded weather data. This worked for practically all districts, even cities with small shares of agriculture. Only five districts in the vicinity of the Alps, the city of Suhl, and the forest-dominated Siegen-Wittgenstein district contained less than 1 km^2 assigned to CLC land use code 211. Class 231 (pastures) was used instead there assuming a uniform interspersal with patches of cropland in the real landscape.

As these preprocessing steps are not part of the algorithm as such a directory with district weather data and the other necessary input files of the Germany example are readily provided (Conradt 2021b). It should be noted that it is not necessary to reproduce the very weather variables contained in these data (mean air temperature, precipitation, and sunshine duration) to run the model in other locations. Any alternative combination of monthly variables would be technically possible.

S3 Workflow along the five programs of ABSOLUT

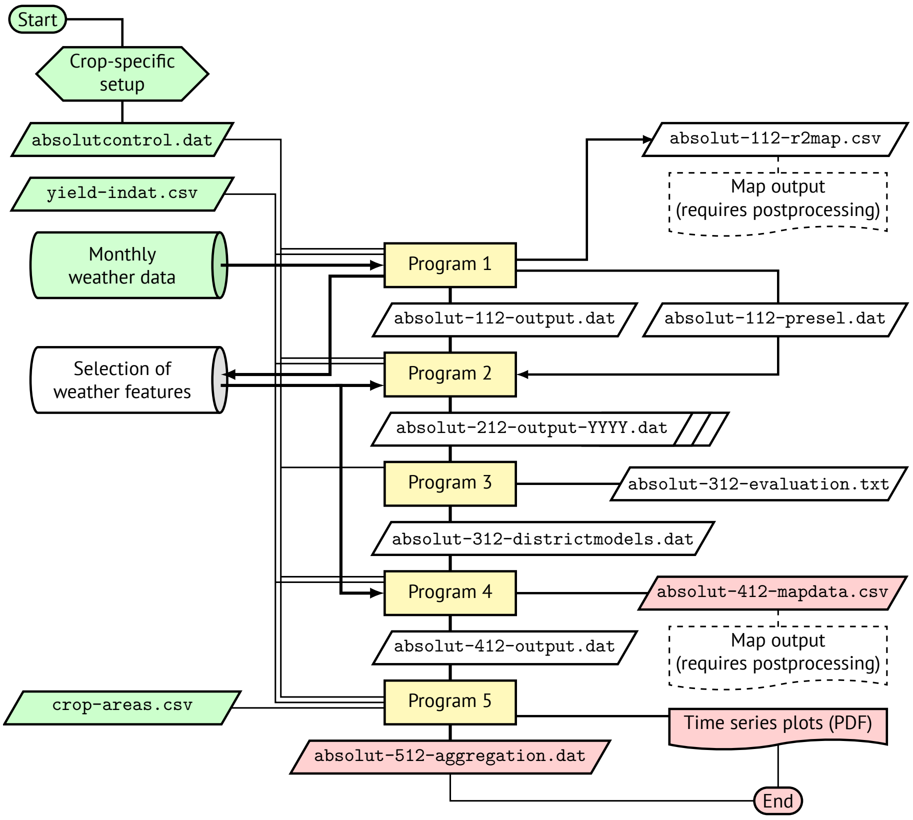

Figure S1: The workflow along the five ABSOLUT programs. Input data are tinted in green, intermediate and pre-check files are symbolized in white, final outputs are tinted in red.

The program code consists of the five R scripts (programs 1–5) which are freely available via Zenodo (Conradt 2021a). Their three-digit numbers consist of one digit for the program number and two digits indicating the version:

```
112_absolut.R
212_absolut.R
312_absolut.R
412_absolut.R
512_absolut.R
```

The required input files (some of which are specific to the Germany test case) can also be downloaded from Zenodo (Conradt 2021b):

absolutcontrol.dat Text file (UTF-8) with case-specific settings for program execution, to be edited by the user before the R scripts are run.

yield-indat.csv Table of crop yields in $dt\,ha^{-1}$ for different crops in German administrative areas, annual values for the years 1999–2020. Each line in this file represents a certain district–year combination and the associated yield observations for different crops. City districts without agriculture must not be included, and the program can also deal with individual data gaps indicated by NA strings.

crop-areas.csv Table of crop areas in hectares for different crops in German administrative areas in the year 2016. The structure is similar to the yield data except that a subdiffentiation by years is (still) missing.

DistrictWeather/ Directory containing district-wise files `dw_01001.dat, dw_01002.dat,...,dw_16077.dat` with monthly weather variables. Each line represents a month in chronological order, and the columns indicate years, months, and the weather variables like temperature, precipitation, etc.

DistrictFeatures/ Directory initially empty.

ClimateScenarios/ Directory containing subdirectories with example climate scenario realisations in the same format as `DistrictWeather/`.

ClimateScenarioFeatures/ Directory initially empty.

The user settings stored in `absolutcontrol.dat` include:

- The principal weather variables to be considered,
- The crop species to be modelled,
- An option to truncate the time periods of weather and yield data input,
- The last month of weather data to be considered before harvest,
- The minimum of yield data years required for a district to be included, and
- The maximum number of different feature combinations selected in program 3.

The flowchart in Figure S1 is a little more detailed and more in line with the technical rules for flowcharts than the version in the scientific article.

The programs 1–5 (code files `112-absolut.R` to `512-absolut.R`) have to be run in sequential order; programs 1 and 2 are only in themselves prepared for parallel processing on a multi-CPU platform. Program 5 is optional, it aggregates the district-wise crop yield predictions and produces time series plots. All code is written under and for Linux systems – Windows users will have to edit file paths (backslashes), system calls, and probably more.

S3.1 Program 1 – "the prospector"

Program 1 loops over all districts for which both climate and yield data are present. The number of considered districts will usually be further reduced by the minimum requirement of yield data for the currently selected crop. Setting this minimum too low will include unreliable regressions while a high setting will unneccessarily throw out a lot of otherwise useful districts; it usually requires balancing between these downsides based on the actual data availability: Longer time series allow for higher thresholds.

This program should be run on as many CPU cores as there are (target) years covered by the input data, because for each of these targets all possible input feature combinations are tested in parallel for their regression fits over the remaining years. (The yields of the target year must be predicted solely from the yield information of the remaining years.) Based on the frequency of weather aggregate features used in the best-performing combinations, features are preselected for each year and stored in `absolut-112-presel.dat`. To enable regression testing, the weather features have to be aggregated beforehand, and they are stored in district files filling the `DistrictFeatures/` directory. At the very end of program 1, the columns in these district files are reduced to the preselected features present in `absolut-112-presel.dat`. The run time of program 1 for the Germany example with two decades of data in 326 districts was about 15 minutes using 24 CPU cores.

The "last month of weather data to be considered" set in the control file should be a calender month towards the end of the average growing season of the crop to be considered. This defines the 12-month periods for the weather feature aggregations.

To exclude single-month features and aggregations over more than six months was a design decision originally motivated by the already high number of features, but testing showed high noise levels for single months and many-month features being only rarely selected.

The weather features are named by combining the meteorological variable acronym with two-digit numbers of the start and end months, thus pr1203 accordingly identifies precipitation in the four months from December to March. For each district considered a file with a wide table of all weather features is stored in a designated directory.

For each district all possible combinations of weather features chosen from the pool generated in the previous step are used as input to multiple linear regressions targeting the available yield data. There are searches for each target year whose yield shall be predicted without prior knowledge of the observed yield which is therefore censored in the process. For each of the

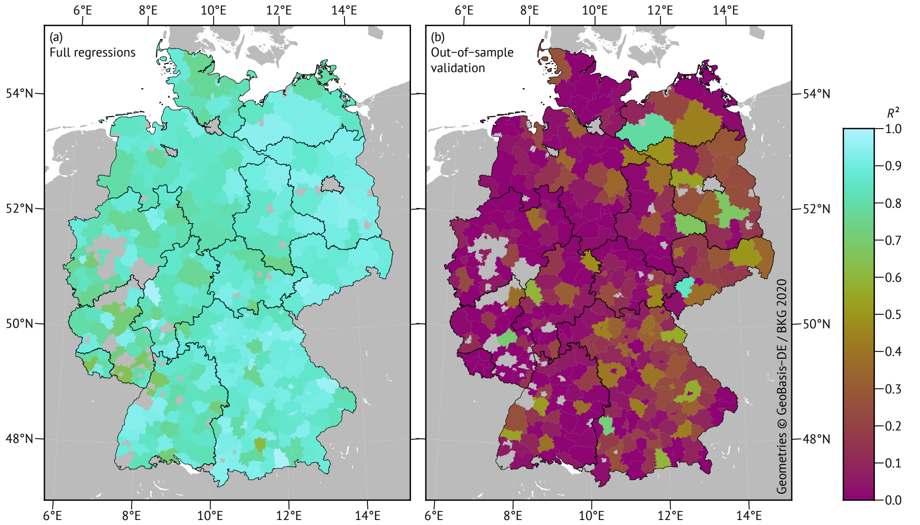

Figure S2: Spatial distributions of squared Pearson correlations (r^2) between predictions and observations for winter wheat yield regressions across Germany for the years 1999–2020. (a) Weather feature selection based on the entire observational data. (b) Predictions made with the same input features but without the actually observed values of the target years used for parameter estimation (leave-one-out validation).

district–target year combinations the 23 best-performing feature combinations – another design decision, 42 would probably work as well – are recorded.

The output is collected in two files: one listing the district IDs, target years, and observed yields (23 times each), followed by out-of-sample yield predictions, coefficients of determination (R^2), and weather feature names of the 23 selected combinations; the other one additionally listing the squared Pearson correlations between predicted and observed district yields for GIS import and mapping.

As already mentioned and evident from Figure S2, the best-fitting feature combinations found here cannot be directly used for reliable yield predictions, because the per-district information is not sufficient to both select a valid combination of regressors and at the same time estimate their coefficients; the textbook calculation of degrees of freedom and standard error only applies if there is no extra freedom in choosing the regressors. This is why the exhaustive search for optimal regressions could be called naïve in the first place.

Another small output file listing the q features for each target year is produced. Finally the district files containing the wide tables of weather features are rewritten, now constrained to the features selected for at least one of the target years as only those will be used in the remaining working steps.

S3.2 Program 2 – "the workhorse"

Since version 1.2, the possible weather feature combinations evaluated in program 2 are not only limited by feature preselection but also by excluding overlapping features of the same weather variable: A combination including precipitation averages for both Janurary–March and February–May will be discarded up-front.

Program 2 should be provided a number of CPU cores which either slightly exceeds the number of districts with sufficient crop yield data – the minimum requirement per district can be set in `absolutcontrol.dat` – or a basic fraction (half, third, etc.) of that number. If, for instance, there are computing nodes of 16 cores each, using seven of them would be a sound decision for the Germany example, because $7 \cdot 16 = 112$ is a bit more than a third of 326. This was actually the setup used by the author, and it took several hours for program 2 to complete. The computational demand is closely related to the number of weather features q selected by target year. The number of feature combinations A is however usually much lower than the combinatorially term on the left hand side of Equation 1 due to the overlap removal.

$$\sum_{d=0}^{4} \binom{q}{d} \geq A \tag{1}$$

For winter wheat q ranged between 29 and 36 which theoretically means up to 66 712 different combinations. Overlap removal left only 4050–15567 allowed combinations for the example years. For each target year, program 2 produces a big table (e. g. `absolut-212-output-2003.dat`) with Pearson correlations of the out-of-sample regression predictions compared to observed yields, for each district (column) and feature (line) combination. A former development version had the table body filled with squared correlations, but these "R^2 values" erroneously indicate predictive power by positive values when there are in fact stronger negative correlations.

S3.3 Program 3 – "the gold pan"

This program selects, based on these tables and separately for each target year, the regressions finally to be used for district yield prediction. This information is collected in `absolut-312-districtmodels.dat`. The other file produced, `absolut-312-evaluation.txt`, is a human-readable ASCII document, originally it had been a diagnostic screen output. The program run time in the Germany example was about 15 minutes on a single CPU.

How many combinations are included from every selection chunk is set via the control file, but the number of different combinations in the merged subset (typically 2–4 times higher) cannot be exactly determined in advance.

There are also alternative, more straightforward selection methods implemented in the code, however deactivated in the distributed version. Methods whose results for the example application are reported in section S4 below are:

Significantly elevated *r*: Inclusion of any combination with a significant shift towards higher-than-average r. This is determined by a rank-based approach; combinations which are significantly overrepresented in a the upper halves, thirds, quarters, and so on (down to five per cent) of the district rankings are globally selected. This may yield several thousand "allowed" feature combinations.

Best global: The subset consists of the combinations with the highest r averages across all districts. The size of the subset is the number given in the control file.

Lowest rank sums: Analogously, a subset with minimal rank sum of the members' per-district correlations is selected, and the same direct control file cutoff applies.

No preselection (control): This is the zero variant for diagnostic purposes. No global preselection is made, so each district is assigned the locally best-performing out of all possible combinations independently from the correlations in other districts.

With the exception of the zero variant, the general principle is always subsetting the A possible combinations to a subset of C "allowed" combinations. As for each district the locally best-performing feature combination from this subset is used, only c combinations will be finally used, and it holds $A > C \geq c$. Once more it must be noted that this is done separately for each target year; this is indicated in the output listing the selected features after the years and district IDs.

S3.4 Programs 4 and 5 – "crucible and mould"

Applying the selected regression equations by program 4 can include yield predictions of the current harvest season using recent weather data and even calculating yield scenarios from climate scenario data. The latter two applications would be based on the equations for the final target year which in turn are based on all available yield observations. As any input feature combination has already been evaluated, part of the calculation may be repetitive, but the complete results are not recorded by program 2.

Program 4 calculates crop yield predictions for each target year using the input features selected. These usually change in most districts between target

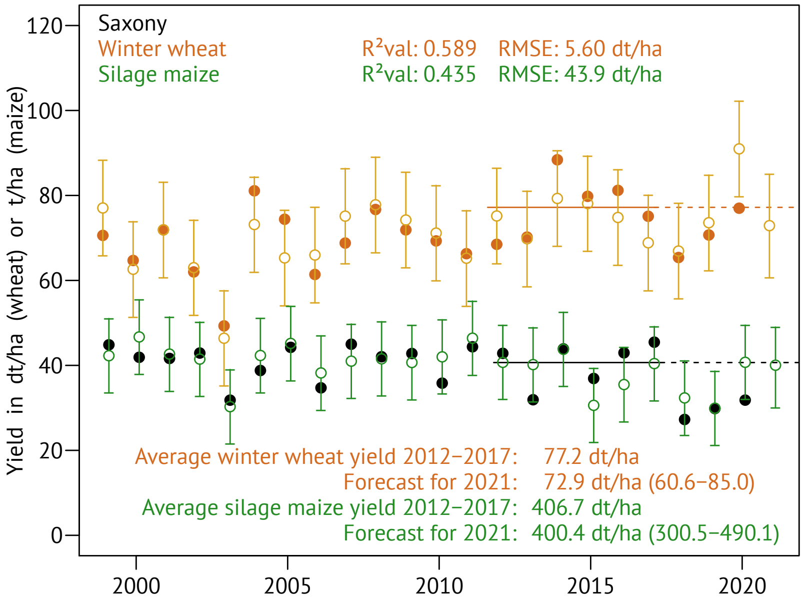

Figure S3: Winter wheat and silage maize yields in the German federal state of Saxony according to the official statistics (DESTATIS 1982ff, solid dots) and their out-of-sample predictions with uncertainty intervals. The uncertainty bars on the hindcasts extend to ±1.96 times the sample standard deviation of the prediction errors s_{err} and should therefore cover 95 % of the observed yields. For 2021 the actual prediction interval is shown, this is slightly wider due to the uncertainty of the s_{err} estimation and centered on the sample mean of prediction errors which may slightly deviate from zero.

years. The test run took about 45 seconds to complete. Program 4 includes the capability for scenario modelling: to activate, you need to edit the code file `412_absolut.R`. Line 32 must be changed to `scenariorun <- TRUE`, and in line 33 a base trend selection can be made.

Program 5 reads the yield predictions on district level and the observed yields of the past plus the file containing the crop areas per district. Currently static areas are assumed, changes of cropping intensities over time might be considered in future program versions. This information is used to aggregate the district yield predictions through weighted averaging to predictions for the full modelling domain – the actual implementaion makes aggregations for federal states and derives national level predictions in a subsequent aggregation step. Among the outputs are time series plots of observations and predictions for the spatial aggregations, Fig. S3 shows an example for the German federal state of Saxony.

Program 5 is not as generally applicable to other modelling domains as the other programs. It is strongly adapted to the German case using a two-stage aggregation from districts via federal states to the national results, with hard-coded two-digit keys of federal states. There are other customizations galore, and users are advised to modify and carefully check this program according to their individual needs.

S4 Sensitivity to the selection alternatives in program 3

Tables S1 and S2 on the following page show results for winter wheat and silage maize obtained for alternative settings in program 3. For the target years 2018, 2019, and 2020 only the yield observations from preceding years were used as input to ensure realistic forecast challenges. For these years, $\mathrm{RMSE_p}$ values indicate the average prediction error across the spatial aggregates (federal states and Germany as a whole). In addition, the complete national time series have been re-estimated. For these, R^2_{val} and $\mathrm{RMSE_g}$ values show the correspondence of the predicted and observed national yield time series (TS). It must be noted that 2018 and 2019 accidentally coincide with a historic drought over Central Europe; the linear extrapolations from less extreme weather and yield fluctuations of the past are probably driven beyond their validity domain.

The most important columns in Tables S1 and S2 are the performance indicators for the national TS predictions to the right, because they integrate all available district results. Clear performance gains can be observed only for the "global and local heroes" approach: R^2_{val} for winter wheat can be increased from 0.259 to more than 0.420, and more than 0.840 can be reached for silage maize. The $\mathrm{RMSE_g}$ are also minimal for both crops using this approach. The picture is less clear for the $\mathrm{RMSE_p}$; compared to the control run without subsetting there are improvements for 2018 winter wheat and 2019 silage maize but also larger errors with 2019 winter wheat and 2018 silage maize. Nevertheless, the "global and local heroes 5" approach seems to be the most preferrable alternative for national yield predictions and was therefore chosen as standard

Table S1: Prediction performances for different approaches in the global preselection of weather aggregate combinations – Winter wheat from 1999 to 2018, 2019 and 2020. The *c*-columns list the numbers of different combinations finally used. The performance indicators R^2_{val} and $RMSE_g$ refer to the time series of national quasi out-of-sample predictions while $RMSE_p$ is calculated from the target-year specific prediction errors in the federal states and Germany.

	Single year forecasts for ...						TS predicitons	
	...2018		...2019		...2020		1999–2020	
Preselection approach	*c*	$RMSE_p$	*c*	$RMSE_p$	*c*	$RMSE_p$	R^2_{val}	$RMSE_g$
No preselection (control)	292	9.18	280	6.12	266	6.14	0.259	5.06
Significantly elevated *r*	267	8.72	253	6.21	253	6.02	0.273	5.00
Best global: Top 100	84	7.36	86	6.25	87	6.22	0.306	4.96
Best global: Top 50	47	6.87	48	6.73	49	6.46	0.323	4.99
Best global: Top 25	25	7.11	25	6.87	25	6.35	0.330	5.00
Best global: Top 15	15	6.84	15	6.76	15	7.22	0.317	5.10
Best global: Top 5	5	6.86	5	6.88	5	7.45	0.290	5.34
Best global: Number 1	1	7.28	1	7.30	1	7.46	0.268	5.45
Rank sums: Bottom 100	79	7.49	82	6.22	83	6.37	0.289	5.06
Rank sums: Bottom 25	25	6.69	25	6.83	25	6.75	0.259	5.22
Rank sums: Bottom 5	5	6.57	5	6.77	5	8.03	0.240	5.66
Global and local heroes 21	46	6.84	43	6.43	45	6.47	0.351	4.86
Global and local heroes 11	32	6.79	30	6.70	22	6.64	0.385	4.72
Global and local heroes 7	22	6.48	23	6.70	15	6.70	0.395	4.69
Global and local heroes 5	16	6.74	17	6.66	13	6.61	0.417	4.58
Global and local heroes 3	10	6.69	5	7.01	8	6.92	0.425	4.52
Global and local heroes 1	4	6.33	2	7.13	4	6.09	0.399	4.71

Table S2: Prediction performances for different approaches in the global preselection of weather aggregate combinations – Silage maize from 1999 to 2018, 2019 and 2020. Column headings as in Table S1.

	Single year forecasts for ...						TS predicitons	
	...2018		...2019		...2020		1999–2020	
Preselection approach	*c*	$RMSE_p$	*c*	$RMSE_p$	*c*	$RMSE_p$	R^2_{val}	$RMSE_g$
No preselection (control)	280	48.2	270	39.3	275	35.8	0.775	16.3
Significantly elevated *r*	238	47.4	197	39.2	237	37.0	0.784	16.0
Best global: Top 100	84	46.6	88	35.7	88	34.9	0.759	16.9
Best global: Top 50	47	49.3	49	31.0	48	34.8	0.771	16.4
Best global: Top 25	25	51.6	25	31.5	25	36.1	0.780	16.1
Best global: Top 15	15	49.1	15	30.1	15	32.8	0.791	15.8
Best global: Top 5	5	51.0	5	30.5	5	30.1	0.789	15.8
Best global: Number 1	1	55.3	1	43.4	1	29.6	0.734	18.2
Global and local heroes 21	41	46.5	50	34.5	47	36.2	0.819	14.6
Global and local heores 11	25	50.3	24	29.8	27	34.2	0.833	14.1
Global and local heroes 7	17	53.2	18	30.2	19	34.9	0.840	13.8
Global and local heroes 5	12	53.0	14	30.2	14	37.6	0.842	13.7
Global and local heroes 3	6	46.0	6	31.6	7	32.5	0.805	15.2
Global and local heroes 1	2	48.6	3	29.4	3	29.8	0.748	17.4

S5 Regional performance: Distibutions of district results

S5.1 Silage maize 2018

Silage maize became increasingly popular as fodder and energy crop over the last decades and is now grown on approximately two million hectares. An average yield of 441.3 dt ha^{-1} was observed in the six pre-drought years 2012–2017 (2018 issue of DESTATIS 1982ff). The lowest yield levels are regularly observed in the federal state of Brandenburg where sandy soils are abundant, 340.3 dt ha^{-1} during the pre-drought period, while the most fertile regions like the Lower Rhine Bay landscape usually report yields above 550 dt ha^{-1}. In 2018 the regional variation expanded because the drought hit hardest in Saxony-Anhalt and the south of Brandenburg where district yields dropped below 200 dt ha^{-1} and some farmers were even confronted with complete failure.

Table S3: Distributional properties of 242 unweighted silage maize district yields predicted for 2018 and their observed counterparts.

	Prediction dt ha^{-1}	Observation dt ha^{-1}
Mean	393.6	372.8
Std devn	92.7	96.2
Maximum	646.6	612.2
Q3	448.5	433.2
Median	405.9	374.3
Q1	344.3	305.3
Minimum	87.5	124.8
Pearson's r	0.680	

Table S3 summarizes the district yield predictions for 2018 and the officially reported statistics of those 242 districts for which both figures were available; except for a certain upward bias and a slightly extended range the distribution of predictions looks convincingly similar to what had been observed. The correlation between predicted and observed district yields is however partly owing to the soil fertility pattern. Moving from absolute yields to relative changes (departures from the average district yields of the years 2012–2017) spoils the picture a bit: The correlation coefficient drops to 0.484 ($n = 223$; 19 of the 242 districts lacked complete observations for 2012–2017).

S5.2 Winter wheat 2019

Winter wheat is Germany's most frequently grown crop covering approximately three million hectares of the agricultural areas. The national yield average of the years 2012–2017 was 79.5 dt ha^{-1} (2018 issue of DESTATIS 1982ff).

The spatial distributions of predicted and reported changes shown in maps (a) and (b) of Fig. S4 are also similar, except that the magnitude of observed yield losses was not captured by the prediction. Outside the region which

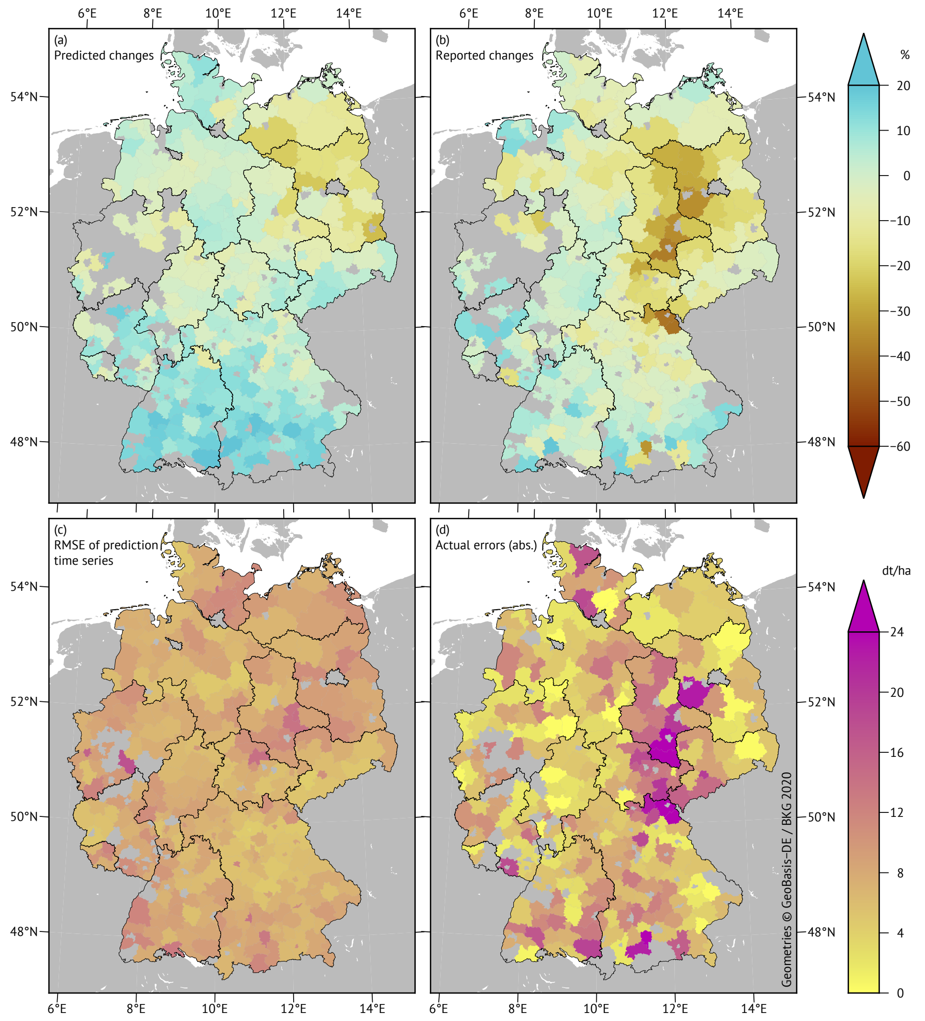

Figure S4: Spatial characteristics of the winter wheat yield prediction for the year 2019. (a) Predicted yield changes compared to the average district yields of the years 2012–2017. (b) Observed changes according to the official statistics. (c) Root-mean-square errors (RMSE) of all out-of-sample district yield predictions for the years 1999–2020. (d) Absolute values of prediction errors for 2019. Panels (a) and (b) show relative deviations in percent (upper scale), (c) and (d) refer to absolute deviations in $dt\,ha^{-1}$ (lower scale).

Table S4: Distributional properties of 280 unweighted winter wheat district yields predicted for 2019 and their observed counterparts.

	Prediction $dt\,ha^{-1}$	Observation $dt\,ha^{-1}$
Mean	79.17	74.03
Std devn	10.98	11.95
Maximum	104.51	103.80
Q3	86.82	82.85
Median	79.65	75.85
Q1	73.27	66.45
Minimum	33.04	39.00
Pearson's r	0.773	

suffered the most extreme yield losses (Fig. S4b) the predictions match quite well contrasting the poor performance for the 2018 silage maize (Fig. S4d).

The distributions of predicted and observed district yields for the year 2019 are shown in Table S4 ($n = 280$). They match reasonably well except a certain general overprediction as observed for 2018 silage maize. The correlation between predicted and observed district yields is a little higher compared to the silage maize example, but for the relative changes to the 2012–2017 yields Pearson's r reaches only 0.460 ($n = 253$).

S6 Error compensation in spatial aggregates

By aggregating district results to regional averages some noise is filtered out by mutual error compensation and error levels diminish. The 2018 silage maize predictions for 242 districts expose an unweighted root-mean-square error (RMSE) of 78.3 dt ha^{-1}, their unweighted mean absolute error (MAE) is 62.9 dt ha^{-1}. The 13 state yield predictions, weighted aggregates of the district results computed with program 5, have unweighted RMSE and MAE values of 54.1 dt ha^{-1} and 49.3 dt ha^{-1}, respectively. The national yield prediction, a weighted aggregate of the state aggregates, missed the official figure by 35.0 dt ha^{-1}.

In the case of the 2019 winter wheat predictions RMSE and MAE of district results ($n = 280$) are at 9.3 and 7.3 dt ha^{-1}, respectively; the state level RMSE and MAE are at 6.8 and 5.3 dt ha^{-1}, respectively; and the national aggregate prediction missed the official national yield by 5.1 dt ha^{-1}. The 2018 and 2019 predictions were generally too optimistic, thus for other years without general biases better error reductions can be expected from spatial aggregations.

Predictions from linear regressions are usually given with prediction intervals based on error estimates. Hence it should theoretically be possible to spatially aggregate these from the district level as well and accordingly give dynamic prediction intervals for state and national predictions for individual years. However, the covariances between the district errors would have to be known for the uncertainty propagation calculation, and this is currently unachievable. The estimated error levels of district predictions hardly correspond to the errors actually observed in single years. Each year exposes a general bias, and in addition to that the district prediction errors are locally correlated.

At the moment, the prediction intervals given for aggregate predictions (the whisker bars in Figs S3) are therefore based on the sample standard deviation of the realized prediction errors. Under certain conditions (extreme weather) errors do however exceed the expected level by far, often concentrated in regional clusters as shown in Figure S4 (d) and Fig. 5d in the main article. The spatial correlation of errors should eventually be further investigated.

S7 Details of the Gornott and Wechsung implementation

The weather variables uniformly used in the STSMs were

- For winter wheat: potential evapotranspiration from November to April and from May to July, temperature normalized radiation from May to July, and precipitation from November to April and from May to July;
- For silage maize: potential evapotranspiration from May to July and from August to October, temperature normalized radiation from May to July, and precipitation from May to July and from August to October.

The potential evapotranspiration had been calculated daily according to Haude (1955), cf. Schrödter (1985). The same holds for the temperature normalized radiation which is solar radiation in $\mathrm{J\,cm^{-2}}$ divided by the average air temperature in °C elevated by 20 to avoid negative denominators. All weather data had been taken from meteorological stations with each district using the data of the one closest to its centre, only in case of multiple stations located within the same district their measurements were averaged (Gornott & Wechsung 2016).

To exclude these peculiar differences in weather data processing from the comparison all weather data are now consistently taken from the monthly DWD grids and preprocessed for the districts as described in subsection S2.2 above. Potential evapotranspiration included in these DWD data is calculated by AMBAV (Löpmeier 2014, Friesland & Löpmeier 2007), very probably closer to reality than the calculations with Haude's formula. Only temperature normalized radiation is not computed on daily basis but using the monthly grids of global radiation and average temperature. The original Gornott & Wechsung (2016) approach used also fertilizer price and acreage of the target crop among their input variables, these are however dismissed here as only the meteorological yield effects are evaluated.

Figures S5 and S6 map the spatial distributions of the out-of-sample accuracies of both approaches for winter wheat and silage maize, resepectively. Practically everywhere the five input variables give only poor results, and the pattern of higher accuracies especially in Northern Germany observed by Gornott and Wechsung (2016) and Conradt et al (2016) is not preserved. At least for silage maize (Fig. S6) the spatial pattern produced by the five variables approach resembles the results of the former studies.

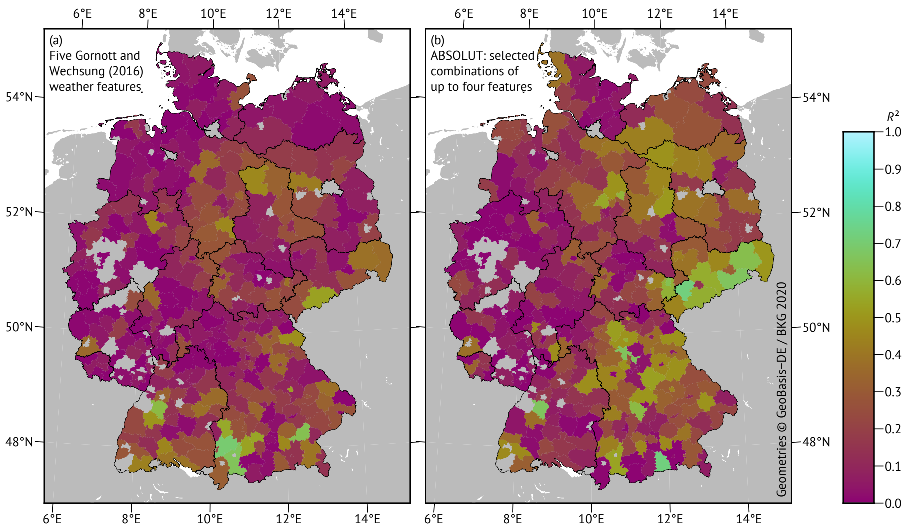

Figure S5: Shares of variance explained by out-of-sample estimations of winter wheat yields on district level: (a) based on the five weather aggregates used by Gornott & Wechsung (2016) and (b) based on individual combinations of four weather aggregates selected through ABSOLUT. The model run of (a) corresponds to the national winter wheat predictions shown in Fig. 5 of the main article and panel (b) to Fig. 4 ibid., respectively.

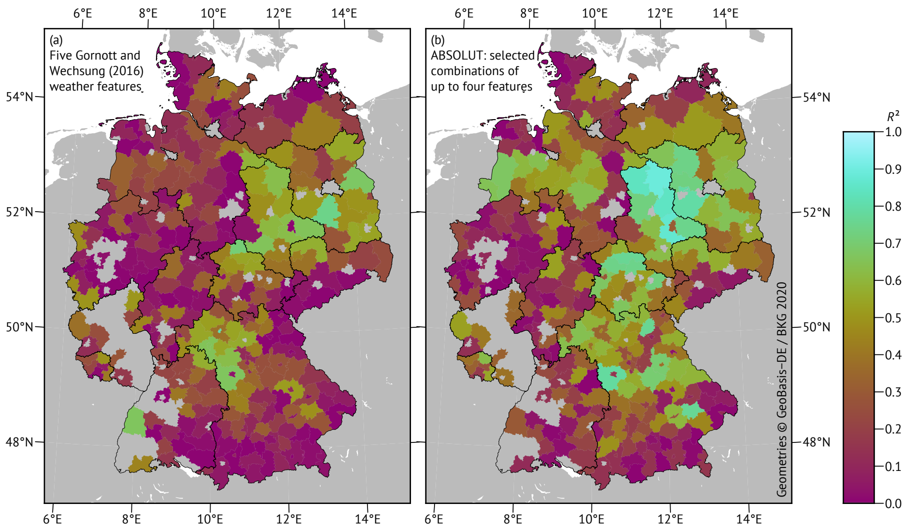

Figure S6: Shares of variance explained by out-of-sample estimations of silage maize yields on district level: (a) based on the five weather aggregates used by Gornott & Wechsung (2016) and (b) based on individual combinations of four weather aggregates selected through ABSOLUT. The model run of (a) corresponds to the national silage maize predictions shown in Fig. 6 of the main article and panel (b) to Fig. 4 ibid., respectively.

References

Conradt, T. (2021a): ABSOLUT R programs (v1.2). Zenodo. doi:10.5281/zenodo.5789350.

Conradt, T. (2021b): ABSOLUT input data for an example application on the districts of Germany (v1.1). Zenodo. doi:10.5281/zenodo.5625774.

Conradt, T. (2021c): The multiple linear regression modelling algorithm ABSOLUT v1.0 for weather-based crop yield prediction and its application to Germany at district level. *Geosci Model Dev Discuss* Preprint: 34 pp. doi:10.5194/gmd-2021-21.

Conradt, T., C. Gornott & F. Wechsung (2016): Extending and improving regionalized winter wheat and silage maize yield regression models for Germany: Enhancing the predictive skill by panel definition through cluster analysis. *Agric For Meteorol* 216: 68–81. doi:10.1016/j.agrformet.2015.10.003.

DESTATIS (1982ff): *Wachstum und Ernte – Feldfrüchte*. Fachserie [Thematic report series] 3, 3.2.1, annual volumes (esp. nos 16), Statistisches Bundesamt, Wiesbaden. URL `https://www.statistischebibliothek.de/mir/receive/DESerie_mods_00000335`. Last access January 2022.

DESTATIS (2017): *Bodennutzung der Betriebe (Landwirtschaftlich genutzte Flächen)*. Fachserie [Thematic report series] 3, 3.1.2, 2016, Statistisches Bundesamt, Wiesbaden. URL `https://www.statistischebibliothek.de/mir/servlets/MCRFileNodeServlet/DEHeft_derivate_00033419/2030312167004_ergaenzt05022018.pdf`. Last access January 2022.

DWD (2020): *Nationaler Klimareport*. Technical Report 4. corrected edn, Deutscher Wetterdienst, Potsdam. URL `https://www.dwd.de/DE/leistungen/nationalerklimareport/download_report_auflage-4.pdf`. Last access January 2022.

European Soil Bureau Network (2005): *Soil Atlas of Europe*. Office for Official Publications of the European Communities, Luxembourg. ISBN 92-894-8120-X, 128 pages. URL `https://esdac.jrc.ec.europa.eu/content/soil-atlas-europe`. Last accessed January 2022.

Friesland, H. & F.-J. Löpmeier (2007): The performance of the model AMBAV for evapotranspiration and soil moisture on Müncheberg data. In: *Modelling water and nutrient dynamics in soil–crop systems*, edited by K. C. Kersebaum, J. M. Hecker, W. Mirschel & M. Wegehenkel, chapter 2, page 19–26. Springer, Dordrecht. doi:10.1007/978-1-4020-4479-3_2.

Gornott, C. & F. Wechsung (2016): Statistical regression models for assessing climate impacts on crop yields: A validation study for winter wheat and silage maize in Germany. *Agric For Meteorol* 217: 89–100. doi:10.1016/j.agrformet.2015.10.005.

Haude, W. (1955): *Zur Bestimmung der Verdunstung auf möglichst einfache Weise*. Mitteilungen des Deutschen Wetterdienstes 11, Deutscher Wetterdienst, Bad Kissingen.

Hennings, V. (2013): Ackerbauliches Ertragspotential der Böden in Deutschland 1 : 1 000 000 (SQR1000). 1 : 1 million map, Bundesanstalt für Geowissenschaften und Rohstoffe, Hannover, Germany. URL `https://www.bgr.bund.de/DE/Themen/Boden/Ressourcenbewertung/`

Ertragspotential/Ertragspotential_node.html. Last accessed January 2022.

Löpmeier, F.-J. (2014): Agrarmeteorologisches Modell zur Berechnung der aktuellen Verdunstung (AMBAV). Online document accompanying the monthly soil moisture grid data on the CDC open data portal of the German Weather Service (DWD), probably republished from the 1983 DWD-internal report Beiträge zur Agrarmeteorologie 7/83. URL https://opendata.dwd.de/climate_environment/CDC/grids_germany/monthly/soil_moist/AMBAV.pdf. Last accessed in January 2022.

Richter, A., C. Siebner, R. Schmidt, G. H. Adler, U. Stegger, W. Eckelmann & R. Hartwich (2007): Nutzungsdifferenzierte Bodenübersichtskarte 1 : 1 000 000 (BÜK1000N). 1 : 1 million map, Bundesanstalt für Geowissenschaften und Rohstoffe, Hannover, Germany. URL https://www.bgr.bund.de/DE/Themen/Boden/Informationsgrundlagen/Bodenkundliche_Karten_Datenbanken/BUEK1000/Nutz_BUEK/nutz_buek_node.html. Last accessed January 2022.

Schrödter, H. (1985): *Verdunstung – Anwendungsorientierte Meßverfahren und Bestimmungsmethoden*. Springer, Heidelberg. ISBN 978-3-540-15355-9, 190 pages.
